# Analysis of Acute Nitrite Exposure on Physiological Stress Response, Oxidative Stress, Gill Tissue Morphology and Immune Response of Large Yellow Croaker (*Larimichthys crocea*)

**DOI:** 10.3390/ani12141791

**Published:** 2022-07-12

**Authors:** Zhenkun Xu, Hongzhi Zhang, Meijie Guo, Dan Fang, Jun Mei, Jing Xie

**Affiliations:** 1 College of Food Science and Technology, Shanghai Ocean University, Shanghai 201306, China; m200300892@st.shou.edu.cn (Z.X.); m200300888@shou.edu.cn (H.Z.); m2103110560@st.shou.edu.cn (M.G.); m210300819@st.shou.edu.cn (D.F.); 2 National Experimental Teaching Demonstration Center for Food Science and Engineering, Shanghai Ocean University, Shanghai 201306, China; 3 Shanghai Engineering Research Center of Aquatic Product Processing and Preservation, Shanghai 201306, China; 4 Shanghai Professional Technology Service Platform on Cold Chain Equipment Performance and Energy Saving Evaluation, Shanghai 201306, China

**Keywords:** acute exposure, gill morphology, immune response, nitrite stress, oxidative stress

## Abstract

**Simple Summary:**

Fish are vulnerable to nitrite stress and display variations in physiologic parameter and stress response. In this study, the effects of nitrite on the stress response of large yellow croaker were evaluated. Large yellow croaker were exposed to four (i.e., 0, 29.36, 58.73, and 88.09 mg/L) nitrite concentrations for 48 h. The results of this study indicated that nitrite exposure could induce oxidative stress in the gills and serum of large yellow croaker. The result of immunoglobulin and lysozyme levels indicated that nitrite exposure could cause an immune response in large yellow croaker. In addition, nitrite stress changed the morphology of the gill of large yellow croaker.

**Abstract:**

Nitrite is a common pollutant in aquaculture water, and nitrite toxicity that negatively affects aquatic species is common in aquaculture systems when the water quality is low. Therefore, the present research aimed to evaluate the effect of acute nitrite exposure on the hematological parameters, antioxidant enzymes, immune response, and gill morphology of large yellow croaker (*Larimichthys crocea*). The fish were randomly separated and exposed to four (i.e., 0, 29.36, 58.73, and 88.09 mg/L) nitrite concentrations for 48 h. The fish blood and gills were collected at 0, 12, 24, 36, and 48 h of nitrite exposure for further analysis. In hematological parameters, the results showed that the levels of hemoglobin, triglyceride, and total cholesterol in blood significantly decreased (*p* < 0.05) in all nitrite-treated samples after 12 h, while the contents of methemoglobin in blood significantly increased (*p* < 0.05) in these treatments. After 48 h of nitrite exposure, the levels of cortisol in serum showed a 94.5%, 132.1%, and 165.6% increase in fish exposed to 29.36, 58.73, and 88.09 mg/L nitrite, respectively. The nitrite (i.e., 29.36, 58.73, and 88.09 mg/L) exposure significantly increased (*p* < 0.05) the levels of antioxidant enzymes (i.e., catalase and glutathione) in the gill and serum after 12 h of exposure compared with the control. The lysozyme levels in serum decreased in the nitrite (i.e., 29.36, 58.73, and 88.09 mg/L) exposure samples. It was found that immunoglobulin levels in the 29.36, 58.73, and 88.09 mg/L nitrite-treated samples (i.e., 1.86, 1.58, and 0.74 μg/mL, respectively) were lower than that of the control (2.56 μg/mL). In addition, the surface of the gill lamellae displayed deformation and contraction after 48 h of nitrite, especially in the fish exposed to 88.09 mg/L nitrite. These results indicate that the nitrite exposure induced the oxidative stress, affected the immune response, and changed the gill morphology, leading to nitrite poisoning in large yellow croaker.

## 1. Introduction

Fresh fish is becoming increasingly important in aquatic product trade [1]. However, the intensification of fish farming causes excessive feeding, higher stocking densities, and unbalanced diets, which have increased the levels of nitrite and other toxic compounds in aquaculture systems. Therefore, fish are vulnerable to aberrant environmental stress factors and display variations in physiologic parameters and stress response [2]. Nitrite is an intermediate toxic product produced by bacteria in the process of ammonia nitrification to nitrate and denitrification [3]. In aquaculture environments, nitrite can easily reach toxic levels and seriously affect the normal growth and health status of aquatic animals [4]. Nitrite bioaccumulates in the blood through the gills, and then enters the red blood cells, where it oxidizes hemoglobin (Hb) to methemoglobin (MetHb) [5]. MetHb reduces the oxygen-carrying capacity of blood under nitrite exposure conditions, leading to hypoxia in fish, which is the main cause of nitrite poisoning in fish [6]. Fish exposed to high levels of nitrite may cause nitrite to accumulate and affect hematological parameters in fish. Thus, hematological parameters are reliable indicators in assessing the toxic effects of nitrite exposure on fish.

In aquaculture environments, nitrite can easily reach toxic levels. For example, the nitrite concentration may accumulate in recirculation aquaculture systems and reach over 50 mg/L NO_2_, even exceeding 50 mg/L NO_2_. This elevated nitrite concentration is toxic to fish. Evidence from many studies suggests that nitrite can interfere with ionic balance and induce oxidative stress in organisms by increasing the generation of reactive oxygen species (ROS) and suppressing the immune response [7,8,9,10,11,12,13]. Antioxidant response can be used as a good indicator to evaluate toxicity in aquatic animals [7]. Fish have many defense mechanisms to adapt to elevated nitrite concentration levels in aquaculture environments. Antioxidant enzymes, such as superoxide dismutase (SOD), catalase (CAT), and glutathione peroxidase (GSH-Px), participate in the process of scavenging excessive ROS, which is one of the mechanisms in fish that counteract oxidative stress [8]. Kim et al. found that nitrite exposure induced an increase in SOD activity in the liver and gills, indicating that nitrite exposure increased the production of ROS and affected antioxidant responses [9]. Zhang et al. also reported that nitrite increased the total antioxidant capacity, CAT activity, GSH-Px activity, and glutathione content with prolonged exposure time [3]. Stress enhancement and the accumulation of oxygen free radicals in the body depress the antioxidant system and directly or indirectly impair the immune viability of the fish. The fish immune system consists of a specific immune system and a nonspecific immune system, and the nonspecific immune system is the first line of defense against stress [10]. Lysozyme (LZM) and immunoglobulin (IgM) play a key role in resisting nitrite stress in nonspecific immunity [11]. Kim et al. reported that LZM and IgM in *P. olivaceus* were significantly stimulated by nitrite exposure, which induces immunogen activation [12].

Fish gill is a multifunctional organ that plays an important role not only in osmotic and ion regulation but also in acid–base regulation and waste excretion [13]. In addition, gill is the primary organ for the absorption and purification of many toxic substances including nitrites [14]. As with the bioaccumulation of contaminants, nitrite accumulates not only in blood plasma but also in gill, liver, and muscle tissue [15]. However, gills are extremely sensitive to toxic chemicals compared to other organs and are more vulnerable to toxic substances than other organs [16]. Studies have found that nitrite exposure can cause damage to the gills of the fish. Therefore, we chose gills as the target tissue.

Large yellow croaker (*Larimichthys crocea*) is an important aquaculture marine fish in China [1,17]. However, high-density recirculating aquaculture results in nitrite concentration rising to a high level in a short time, thereby threating the survival of large yellow croaker. At present, studies on the toxic effects of nitrite on fish are mainly focused on the effects of nitrite on fish growth and survival [18]. However, acute exposure to nitrite concentrations beyond their protective capacity can also lead to poisoning of aquaculture animals [19]. Comprehending the acute toxicity of nitrite exposure is essential for knowing the physiology and tolerance threshold of fish. Therefore, the purpose of this research was to investigate the possible mechanisms of the toxic response to nitrite exposure in large yellow croaker by examining hematological and serum biochemical parameters, oxidative stress, and immune responses following acute nitrite stress in large yellow croaker.

## 2. Materials and Methods

### 2.1. Fish and Experimental Conditions

The experiment procedure was approved by the Animal Care and Use Committee of the Shanghai Ocean University (SHOU-DW-2021-72). Large yellow croaker (weight: 600 ± 50 g, *n* = 576) of 8 months of mean age were purchased from a local seafood market in Luchao Port town (Shanghai, China). All fish were acclimated in a 400 L polyethylene tank with seawater to the experimental conditions for 2 days. All fish were fasted over the acclimation period. During the acclimation and test periods, the water temperature was maintained at 20 °C, the water salinity was kept at 20%, the pH value was kept at 7.5, and the concentration of dissolved oxygen (DO) was higher than 7.0 mg/L. One-third of the water was replaced daily to ensure good water quality, according to the method of Lin et al. [20]. The water was recycled through a recirculation filter.

### 2.2. Experimental Design

The 48 h LC50 concentration of nitrite for large yellow croaker was determined to be 97.88 mg/L according to the research method of Lin et al. [16]. The nitrite concentrations were chosen according to the study by Lin et al. [20], with minor modifications. Based on the LC50 dose, the low concentrations of nitrite (30%, 48 h, LC50), medium concentrations of nitrite (60%, 48 h, LC50), and high concentrations of nitrite (90%, 48 h, LC50) were chosen to evaluate the effect of acutely exposed nitrite on large yellow croaker. After 2 days of acclimation, 360 fish were randomly chosen for a 48 h acute exposure to nitrite at 4 concentrations: 29.36 (low), 58.73 (medium), 88.09 (high), and 0 mg/L (control). There were 30 fish in each group, and the experiments were conducted in triplicate. The required nitrite levels were reached by adding nitrite test solutions (10 g/L), or seawater was added every 12 h. Nitrite test solutions (10 g/L) were prepared using sodium nitrite (purity ≥ 99.96%, Aladdin Biotechnology Co., Ltd., Xi’an, China) in seawater. Four groups were used in this study. The samples were labeled: NO-29.36, NO-58.73, NO-88.09, and CK, respectively. CK, NO-29.36, NO-58.73, and NO-88.09 represented 0, 29.36, 58.73, and 88.09 mg/L nitrite concentrations, respectively. The waterborne nitrite levels were measured based according to the method of Lin et al. [20].

### 2.3. Sample Collection

Three fish from each group were randomly sampled at the 0, 12th, 24th, 36th, and 48th h of nitrite exposure. On the one hand, the blood of large yellow croaker was collected from the tail vein into heparinized syringes for further analysis. On the other hand, the blood of large yellow croaker was taken from the tail vein without anticoagulant, stored at 4 °C for 12 h, and then centrifuged at 3000× *g* at 4 °C for 20 min, and the liquid supernatant (serum) was collected and stored at −80 °C before use. The gills of each fish were removed and stored at −80 °C until further analysis. The gill filament of large yellow croaker was immediately removed for immersion in a 2.5% concentration of glutaraldehyde solution for 24 h until further analysis.

### 2.4. Blood Biochemical Index Determination

Three fish from each group were randomly sampled at the 0, 12th, 24th, 36th, and 48th h of nitrite exposure. The Hb concentration in the blood was determined with the method of Zhang et al. [21] while the MetHb was analyzed according to the method of Wuertz et al. [22]. The serum concentrations of triglyceride (TG), and total cholesterol (TC) were measured using commercial kits (Nanjing Jiancheng Bioengineering Institute, Nanjing, China). The levels of cortisol were measured using the antibody-antigen-enzyme-antibody complex, and then the absorbance of the solution was measured at 450 nm.

### 2.5. Antioxidant Response Analyses

For antioxidant response analyses, three fish from each group were randomly sampled at the 0, 12th, 24th, 36th, and 48th h of nitrite exposure. Serum and gill were sampled at the 0, 12th, 24th, 36th, and 48th of nitrite exposure. The gill tissue (0.1 g) was washed with 0.85% precooled physiological saline and homogenized in an ice bath with 0.9 mL physiological saline. The mixture was then centrifuged at 3000× *g* for 15 min at 4 °C, and the supernatant was immediately retransferred to determine the activity of SOD, CAT, and GSH-PX. The blood was centrifuged at 3000× *g* at 4 °C for 20 min, and the liquid supernatant (serum) was collected to determine the activities of SOD, CAT, and GSH-PX.

### 2.6. Immune Responses Analysis

For immune responses analyses, three fish from each group were randomly sampled at the 0, 12th, 24th, 36th, and 48th h of nitrite exposure. The IgM level in the fish was measured using the antibody–antigen–enzyme–antibody complex. The absorbance of the solution was measured at 450 nm. The LZM activity analysis was measured following the method by Gao et al. [10].

### 2.7. Histological Analysis

For histological responses analyses, three fish from each group were randomly sampled at the 0, 12th, 24th, 36th, and 48th h of nitrite exposure. The upper gill filaments of the second gill arch of large yellow croaker were cut and treated using the method of Wang et al. [23]. The gill filament for the second gill arch of large yellow croaker were fixed in a 2.5% concentration of glutaraldehyde solution for 24 h, and then washed with 0.1 mol/L phosphate buffer (pH = 7.4) 3 times. Gradient elution with 30, 50, 70, 80, 90, and 100% ethanol was performed for 15 min and then placed in a vacuum freeze dryer (Minifast 04, Shanghai, China) for 12 h. The morphology of gill tissues samples was observed by scanning electron microscopy (SEM) SU5000 (Hitachi, Tokyo, Japan). According to the method of Nie et al. [24], imaging software was used to measure the interlaminar distance and lamellae surface area on gill of large yellow croaker.

### 2.8. Statistical Analyses

Statistical analyses were performed using IBM SPSS Statistics version 18.0 software. The normality of all data were checked using the Kolmogorov–Smirnov test. The isolated and interactive effects of exposure time and nitrite treatment were analyzed using a two-way ANOVA (listed in Table 1) according to the method of Hosseini et al. [25]. If significant differences of interaction were found, Duncan’s multiple range tests were used to determine the differences between means. The significance level was set to *p* < 0.05. Experimental data are expressed as the mean ± standard deviation (SD) [26].

## 3. Results

### 3.1. Effect of Nitrite Exposure on Hb and MetHb

**Figure 1:**
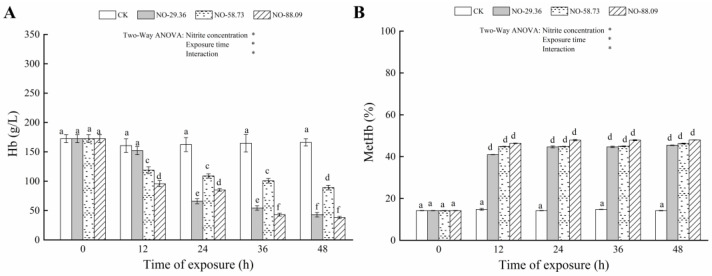
Changes in hemoglobin (Hb) (**A**) and methemoglobin (MetHb) (**B**) concentrations in the blood of large yellow croaker exposed to nitrite at 29.36 mg/L (NO-29.36), 58.73 mg/L (NO-58.73), 88.09 mg/L (NO-88.09), and 0 mg/L (CK) for 48 h. Values are expressed as the mean ± SD. Different lowercase letters indicate significant differences (*p* < 0.05) among groups. The CK nitrite group served as the control. * *p* < 0.05.

Compared to the control group, the Hb content significantly decreased (*p* < 0.05) in fish exposed to 58.73 and 88.09 mg/L nitrite for 12 h (Figure 1A). Similar results were observed in fish treated with 29.36 mg/L nitrite for 24 h (*p* < 0.05) (Figure 1A). Conversely, fish exposed to 29.36, 58.73, and 88.09 mg/L nitrite showed increased MetHb levels after 12 h that were significantly higher than the control group (*p* < 0.05) (Figure 1B).

### 3.2. Effect of Nitrite Exposure on the Serum Biochemical Index

**Figure 2:**
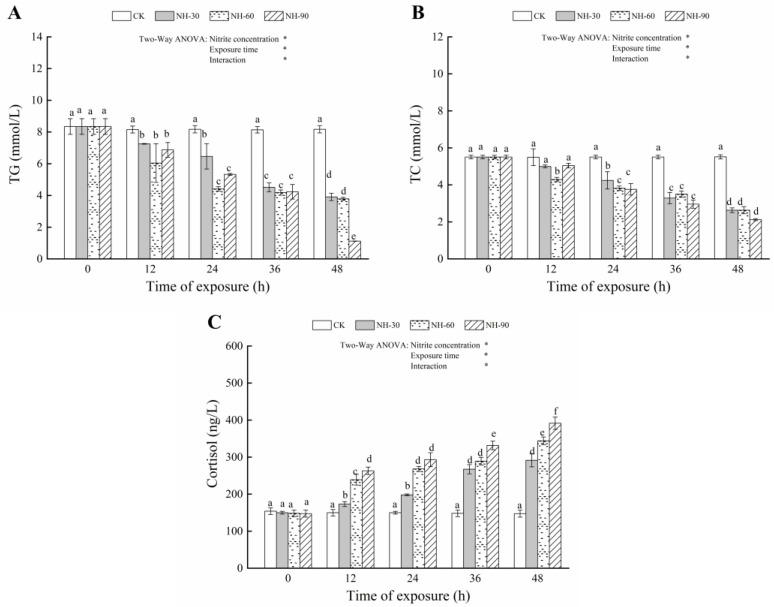
Changes in triglyceride (TG) (**A**), total cholesterol (TC) (**B**), and cortisol (**C**) levels in the serum of large yellow croaker exposed to nitrite at 29.36 mg/L (NO-29.36), 58.73 mg/L (NO-58.73), 88.09 mg/L (NO-88.09), and 0 mg/L (CK) for 48 h. Values are expressed as the mean ± SD. Different lowercase letters indicate significant differences (*p* < 0.05) among groups. The CK nitrite group served as the control. * *p* < 0.05.

The TG and TC levels in the treatments with 29.36, 58.73, and 8.09 mg/L nitrite for 24, 36, and 48 h were significantly lower (*p* < 0.05) than the control (Figure 2A,B). The decreases in both parameters were also observed when fish were exposed to 58.73 mg/L nitrite for 12 h (*p* < 0.05) (Figure 2A,B). Compared with the controls, exposure to 29.36, 58.73, and 8.09 mg/L nitrite caused marked increases in cortisol levels (*p* < 0.05) (Figure 2C), and the degree of variation in cortisol levels was positively correlated with the nitrite exposure concentration and exposure time.

### 3.3. Effect of Nitrite Exposure on the Activity of Antioxidant Enzymes

**Figure 3:**
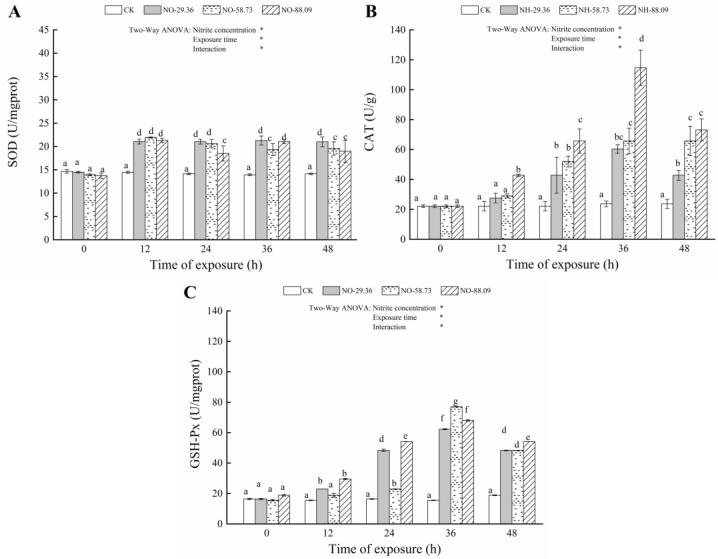
Changes in superoxide dismutase (SOD) (**A**), catalase (CAT) (**B**), and glutathione peroxidase (GSH-Px) (**C**) levels in the gill of large yellow croaker exposed to nitrite at 29.36 mg/L (NO-29.36), 58.73 mg/L (NO-58.73), 88.09 mg/L (NO-88.09), and 0 mg/L (CK) for 48 h. Values are expressed as the mean ± SD. Different lowercase letters indicate significant differences (*p* < 0.05) among groups. The CK nitrite group served as the control. * *p* < 0.05.

**Figure 4:**
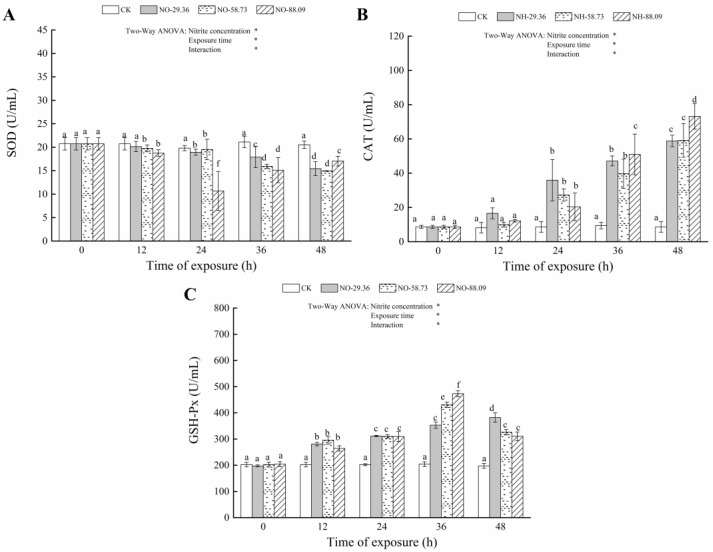
Changes in superoxide dismutase (SOD) (**A**), catalase (CAT) (**B**), and glutathione peroxidase (GSH-Px) (**C**) levels in the serum of large yellow croaker exposed to nitrite at 29.36 mg/L (NO-29.36), 58.73 mg/L (NO-58.73), 88.09 mg/L (NO-88.09), and 0 mg/L (CK) for 48 h. Values are expressed as the mean ± SD. Different lowercase letters indicate significant differences (*p* < 0.05) among groups. The CK nitrite group served as the control. * *p* < 0.05.

Compared with the CK, the SOD activities in gill showed a marked increase when fish were exposed to 29.36, 58.73, and 88.09 mg/L nitrite for 12, 24, 36, and 48 h (*p* < 0.05) (Figure 3A). Conversely, the SOD activities in the serum significantly decreased (*p* < 0.05) after exposure to 58.73 and 88.09 mg/L nitrite for 12, 24, 36, and 48 h (Figure 4A). A similar decrease in the SOD activity level of serum was observed after exposure to 29.36 mg/L of nitrite for 24 h (*p* < 0.05) (Figure 4A). The CAT activities in the gills significantly increased (*p* < 0.05) after 12 h of exposure to 88.09 mg/L nitrite compared to the control group. Similar results were found in fish treated with 29.36 and 58.73 mg/L of nitrite for 24 h (*p* < 0.05) (Figure 3B). The activities of CAT in serum were also significantly increased (*p* < 0.05) after 24 h of exposure to 29.36, 58.73, and 88.09 mg/L nitrite compared to the control group (Figure 4B). Compared with the CK, the GSH-PX activities in the gill and serum of large yellow croaker significantly increased (*p* < 0.05) after exposure to 29.36, 58.73, and 88.09 mg/L nitrite for 12, 24, 36, and 48 h (Figure 3C and Figure 4C). However, the GSH-PX activities in the gill and serum showed a decrease when fish were exposed to 58.73 and 88.09 mg/L nitrite for 48 h (Figure 3C and Figure 4C).

### 3.4. Effect of Nitrite Exposure on Immune Responses

**Figure 5:**
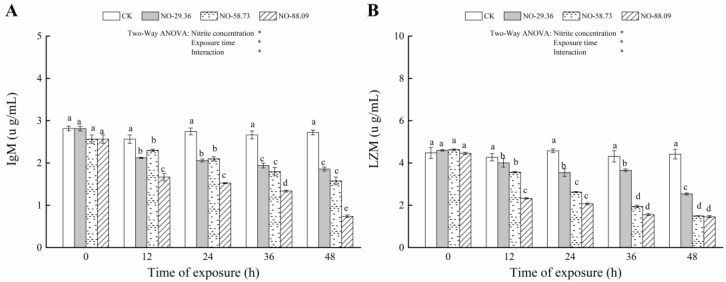
Changes in immunoglobulin (IgM) (**A**) and lysozyme (LZM) (**B**) levels in the serum of large yellow croaker exposed to nitrite at 29.36 mg/L (NO-29.36), 58.73 mg/L (NO-58.73), 88.09 mg/L (NO-88.09), and 0 mg/L (CK) for 48 h. Values are expressed as the mean ± SD. Different lowercase letters indicate significant differences (*p* < 0.05) among groups. The CK nitrite group served as the control. * *p* < 0.05.

The IgM and LZM levels increased (*p* < 0.05) in the serum of large yellow croaker after 12, 24, 36, and 48 h of continuous exposure to 29.36, 58.73, and 8.09 mg/L of nitrite compared to that of the controls (Figure 5A,B). In addition, the change degree of the LZM level was negatively correlated with the nitrite exposure concentration and exposure time (Figure 5B).

### 3.5. Effect of Nitrite Exposure on Gill Morphology

**Figure 6:**
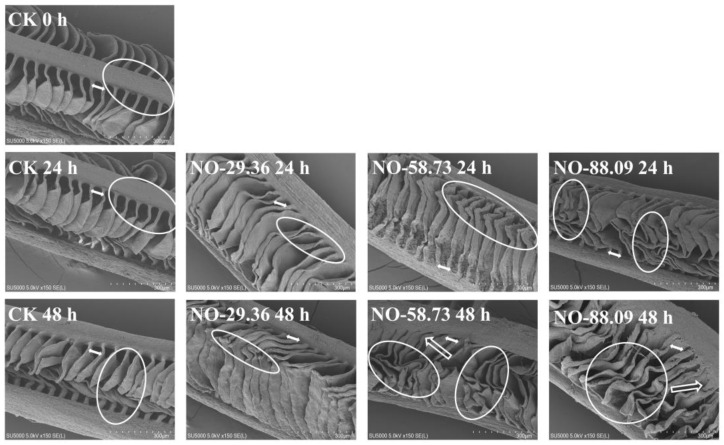
Scanning electron microscope (SEM) micrographs of large yellow croaker gills exposed to nitrite at 29.36 mg/L (NO-29.36), 58.73 mg/L (NO-58.73), 88.09 mg/L (NO-88.09), and 0 mg/L (CK) for 0, 24, and 48 h. Bars = 300 μm. Whitehead arrows designated long lamellae separated by interlamellar spaces.

**Figure 7:**
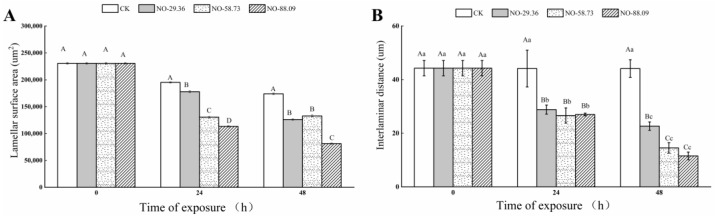
Quantification of fish gill structure. Lamellar surface area of gill (**A**) and interlaminar distance of gill (**B**) in large yellow croaker. Different capital letters indicate significant differences in the means between treatment groups. Different lowercase letters indicate significant differences in the means between the same treatment groups (*p* < 0.05).

The gill lamellae were separated by regular interlamellar spaces (Figure 6, CK 0 h) and did not display significant changes in the surface structure of gills at 24 and 48 h of nitrite exposure in CK (Figure 6, CK 24 h and CK 48 h). The gill lamellae separated by interlamellar intervals were still observed at 24 h of nitrite exposure in NO-29.36; however, the surface of the gill lamellae also displayed a slight shrinkage at 24 h (Figure 6, NO-29.36 24 h). After 48 h of nitrite exposure, the interlaminar distance between the gill lamellae in NO-29.36 became shorter (Figure 6, NO-29.36 48 h). Compared with 24 h of nitrite exposure in NO-58.73, the gill lamellae after 48 h of nitrite exposure not only displayed serious deformation and contraction but also shorter interlaminar distance (Figure 6, NO-58.73 48 h). More severe deformation and contraction were observed in NO-88.09. The interlaminar distance between the gill lamellae became shorter and almost fused at the 48th h in NO-88.09 (Figure 6, NO-88.09 48 h). In addition, gill lamellae in NO-58.73 and NO-88.09 seemed thicker and more curved and irregular than those of NO-29.36 and CK. Moreover, the interlaminar distance and lamellae surface area of large yellow croaker gill were measured. The interlaminar distance and lamellae surface area in all nitrite-treated samples showed a decreasing trend with the extension of exposure time compared with the CK (Figure 7A,B).

## 4. Discussion

Nitrite diffuses from the blood into red blood cells, where it easily oxidizes iron in Hb to form MetHb [6]. In the current study, Hb levels were significantly reduced and MetHb levels increased in all nitrite-treated samples. Meanwhile, we observed a decrease in the concentration of Hb and an increase in the concentration of MetHb with the increase in nitrite concentration and exposure time. These findings suggest that nitrite caused oxidation of Hb to MetHb, reduced the oxygen-carrying capacity of the blood, and caused major toxic effects in the blood of large yellow croaker. Our results supported the previous finding that elevated nitrite levels and exposure times decreased Hb level as well as increased MetHb levels in *Takifugu rubripes* [27].

Lipids can provide energy to meet energy needs and support development in fish [21]. TG and TC play a vital role in fish lipid metabolism. However, nitrite exposure has been reported to disturb lipid metabolic balance. Jia et al. [28] found that a significant increase in TG was observed in juvenile turbot exposed to 0.4 and 0.8 mM nitrite. The mobilization of lipid reserves to meet the increased energy requirements in response to nitrite stress in juvenile turbot may account for this result. However, the present result showed that the TG levels significantly decreased in all nitrite-treated samples after 12 h, indicating that lipid homeostasis in fish was disturbed by nitrite exposure. TC is a critical substance in lipid metabolism and all steroid hormone precursors [29]. In this study, the serum TC level decreased significantly after 12 h of nitrite exposure, indicating that excessive energy was consumed in the resistance to nitrite stress. In addition, Kim et al. [30] suggested that TC level in plasma of *Paralichthys olivaceus* were decreased by exposure to nitrite, which may have inhibited steroid hormone synthesis.

Cortisol is considered a stress indicator in fish stress responses caused by exposure to toxic substances [31]. The serum cortisol increased significantly in all nitrite-treated samples treated for 12 h, indicating that the hypothalamic–pituitary–interrenal (HPI) axis, a typical stress response, was activated in response to nitrite exposure. In addition, nitrite exposure leads to elevated levels of MetHb, leading to hypoxia in fish, and may also be responsible for a significant increase in the stress marker cortisol. Jia et al. [28] also found that the cortisol levels of juvenile turbot in 0.4 and 0.8 mM nitrite-treated samples increased significantly after 48 h of exposure.

Continuous nitrite exposure could lead to oxidative stress in fish, which is accompanied by the production of excessive ROS. Fish have established an antioxidant defense system, including antioxidant enzymes and antioxidants, to protect them from the adverse effects of ROS and oxidative damage [8]. Antioxidant enzymes, such as SOD, CAT, and GSH-Px, in fish act as a key role in balancing between ROS generation and clearance. SOD converts oxygen free radicals in cells into hydrogen peroxide (H_2_O_2_). Subsequently, CAT and GSH-Px converts H_2_O_2_ into H_2_O and O_2_ and reduces its toxic effects [32].

The nitrite-treated samples significantly decreased the SOD activity in serum after exposure to nitrite and significantly increased the SOD activity in gill after exposure to nitrite, indicating that SOD in gill was quickly activated to reduce oxidative damage, while SOD in serum was inhibited. This also indicated that SOD in gill played a greater role in scavenging excessive ROS induced by nitrite exposure than SOD in serum. In this study, the levels of CAT and GSH-Px significantly increased in fish exposed to nitrite, indicating that the fish protected itself from oxidative damage by elevating CAT and GSH-Px levels in gill and serum. However, GSH-Px levels in gills and serum decreased when fish were exposed to 58.73 and 88.09 mg/L of nitrite for 48 h, suggesting that CAT and GSH-Px may not be effective in removing excessive ROS caused by nitrite exposure. It is noteworthy that CAT activity in gills decreased when fish were exposed to 88.09 mg/L of nitrite for 48 h compared with that in serum, which may be caused by serious oxidative damage, such as degradation of antioxidant enzymes in gills, resulting from prolonged exposure time and increased nitrite concentration.

IgM, the main immunoglobulin in fish, are vital molecules in mediating humoral immune responses [33]. The present study found that a significant decrease in serum IgM occurred in fish treated with nitrite for 12 h compared with that of the CK, indicating that nitrite exposure suppressed the expression of IgM protein and interfered with the immune response of large yellow croaker. Zhang et al. [21] also found that yellow catfish treated with 3 and 30 mg/L of nitrite caused a reduction in serum IgM. LZM plays a vital role in the innate immune system of fish. Studies have shown that LZM is a key nonspecific immune protein in mediating defense against microbial invasion [34]. Wang et al. [35] suggested that nitrite exposure could impair the immune capacity in fish. Our results showed that a decrease in the level of LZM with the increase in nitrite concentration and exposure time, further supporting that a high level of nitrite exposure negatively affected immune capacity defense against bacterial diseases. Gao et al. [10] also found that the level of LZM in *Takifugu rubripes* considerably decreased in fish exposed to 3 and 6 mM level of nitrite for 48 and 96 h.

The change in gill morphology may be a protective strategy to adapt to the nitrite stress. The deformation and contraction of gill filaments resulted in an increased diffusion distance between nitrite and the gill vascular system, which inhibited the entry rate of toxic substances into the bloodstream. In addition, nitrite enters the bloodstream through the gills during nitrite exposure, leading to elevated MetHb levels that lead to nitrite poisoning in fish [14]. The result that the interlaminar distance between the gill lamellae became shorter and almost fused also tended to reduce the respiratory surface area, which made the fish less exposed to toxic substances such as nitrite. The results showed that the effect of nitrite on the lamellae surface area of large yellow croakers was realized by reducing the gill lamellae surface area. In the study by Koca et al., a significant decrease in the mean length of primary and secondary lamellae were observed after *Lepomis gibbosus* were exposed to nitrite [36]. Moreover, secondary lamellae fusion, ballooning degenerations, or club deformation of secondary lamellae of Lepomis gibbosus were also observed [36]. These results are similar to our results, indicating that nitrite may affect the gill morphology of fish.

## 5. Conclusions

**Figure 8:**
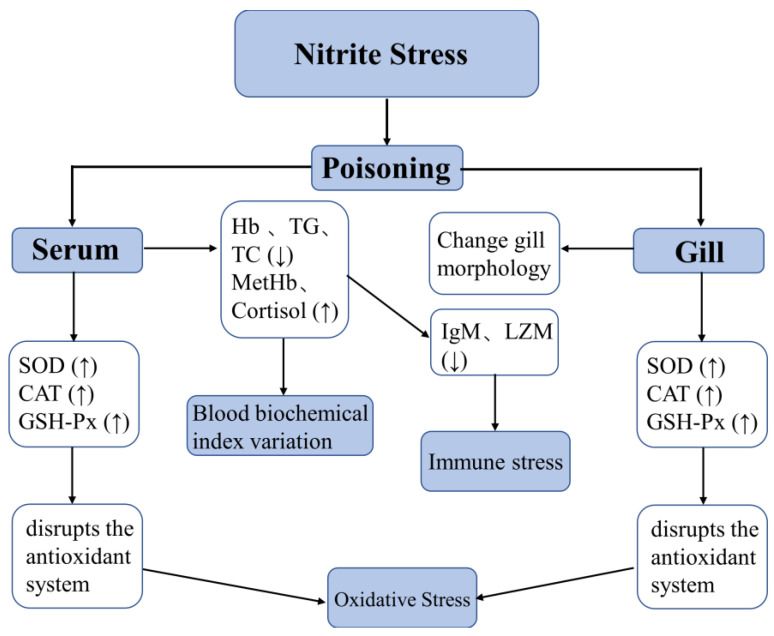
The graphical abstract of the nitrite-induced toxic effect in large yellow croaker via gill and blood. ↑: increase, ↓: decrease.

The effects of acute exposure to nitrite (i.e., 29.36, 58.73, and 88.09 mg/L) on hematological status, oxidative stress, immune response, and gill morphology of large yellow croaker were studied. The results showed that nitrite exposure could reduce the levels of Hb, TG, TC, IgM, and LZM in fish blood and increase levels of MetHb and cortisol, which had toxic effects on fish blood. At the same time, nitrite exposure could also lead to the increase in SOD, CAT, and GSH-Px contents in gills and serum of fish, resulting in oxidative stress in fish. In addition, nitrite exposure also changed the gill morphology including the deformation and contraction of gill lamellae surface. These changes have been observed in the fish exposed to 29.36 mg/L and became more pronounced with prolonged exposure and higher nitrite concentrations. Therefore, it is recommended that the nitrite concentration not exceed 29.36 mg/L in order to protect the fish from oxidative stress and immune stress. Additionally, we showed the graphical abstract of nitrite-induced toxic effect in large yellow croaker via gill and blood (Figure 8).

## Figures and Tables

**Table 1:** The results of the interaction between time and/or nitrite exposure on the levels of hematological biochemical parameters, the activity of antioxidant enzyme, and the immune-related indicator using the two-way ANOVA method are shown below.

Parameters	Source of Variation	df	F	*p*
Hb	Time	4	331.219	<0.01
	Ammonia exposure	3	383.852	<0.01
	Time × Ammonia exposure	12	50.991	<0.01
MetHb	Time	4	517.678	<0.01
	Ammonia exposure	3	928.468	<0.01
	Time × Ammonia exposure	12	58.302	<0.01
TG	Time	4	155.078	<0.01
	Ammonia exposure	3	147.552	<0.01
	Time × Ammonia exposure	12	23.049	<0.01
TC	Time	4	362.718	<0.01
	Ammonia exposure	3	312.93	<0.01
	Time × Ammonia exposure	12	43.642	<0.01
Cortisol	Time	4	298.873	<0.01
	Ammonia exposure	3	438.299	<0.01
	Time × Ammonia exposure	12	45.041	<0.01
SOD (Gill)	Time	4	67.909	<0.01
	Ammonia exposure	3	114.54	<0.01
	Time × Ammonia exposure	12	10.756	<0.01
CAT (Gill)	Time	4	110.384	<0.01
	Ammonia exposure	3	132.469	<0.01
	Time × Ammonia exposure	12	19.899	<0.01
GSH-Px (Gill)	Time	4	1154.843	<0.01
	Ammonia exposure	3	870.060	<0.01
	Time × Ammonia exposure	12	179.636	<0.01
SOD (Serum)	Time	4	13.573	<0.01
	Ammonia exposure	3	16.741	<0.01
	Time × Ammonia exposure	12	5.581	<0.01
CAT (Serum)	Time	4	109.931	<0.01
	Ammonia exposure	3	62.838	<0.01
	Time × Ammonia exposure	12	14.040	<0.01
GSH-Px (Serum)	Time	4	711.795	<0.01
	Ammonia exposure	3	725.022	<0.01
	Time × Ammonia exposure	12	125.188	<0.01
IgM	Time	4	343.445	<0.01
	Ammonia exposure	3	719.004	<0.01
	Time × Ammonia exposure	12	49.992	<0.01
LZM	Time	4	435.107	<0.01
	Ammonia exposure	3	718.365	<0.01
	Time × Ammonia exposure	12	71.034	<0.01

## Data Availability

All data, models, and code generated or used during the study appear in the submitted article.
